# Specificity of eccentric hamstring training and the lack of consistency between strength assessments using conventional test devices

**DOI:** 10.1038/s41598-021-92929-y

**Published:** 2021-06-28

**Authors:** Hans-Peter Wiesinger, Manuel Scharinger, Alexander Kösters, Christoph Gressenbauer, Erich Müller

**Affiliations:** Department of Sport and Exercise Science, University of Salzburg, Schlossalle 49, 5400 Hallein/Rif, Salzburg, Austria

**Keywords:** Physiology, Risk factors

## Abstract

Hamstring injuries are endemic, but influences of test-specific training and the application of different test methods on decision making remain elusive. Sport-students were randomised to isokinetic (IG) or Nordic hamstring (NG) exercise or a control group (CG) for six weeks. Training and testing procedures were matched to biomechanical parameters. Hamstring strength (EPT), work, muscle soreness (visual analogue scale (VAS)), biceps femoris (BF_lh_) muscle size and architecture were assessed. Anthropometrics and strength parameters did not differ at baseline. Yet, body mass normalised EPT, and work revealed a significant group × time × device effect, with a significant main effect for devices. Experimental conditions triggered meaningful increases in EPT compared to the control group, but the effects were higher when recorded on the training device. Despite significant group × time interactions, normalised average work on the NHD was only higher in the NG compared to CG of the left leg (+ 35%). No effects were found for BF_lh_ parameters. Hamstrings showed a high training specificity, but adaptations likely remain undetected owing to the low sensitivity of conventional test devices. Moreover, strength increase of ~ 15% does not necessarily have to be reflected in BF_lh_ parameters.

## Introduction

Posterior thigh muscle injuries are ubiquitous in elite and recreational sports, and a reduction of modifiable risk factors is critical for performance^1^, health^2^, and economic benefit^3^. In recent years, two significant parameters have emerged in this respect, namely a high eccentric knee flexor strength and a long fascicle length^4,5^.

To identify effective yet efficient exercises, researchers analysed the effects of short-term (3–20 weeks) hamstring exercises on muscle activation patterns^6–11^, changes in biceps femoris long head (BF_lh_) fascicle length^12–17^, and reported almost systematic increases in knee flexion strength^14,18–21^. These results have occasionally been accompanied by a shift of the peak torque angles towards a longer muscle length^21,22^. Some of the exercises have also been shown to reduce hamstring strains^23,24^, but the impact of loading stimuli (e.g., training intensity, volume, contraction modality) or the mechanisms of hamstring injury (e.g., contraction modality, fatigue state, intra- and intermuscular coordination) remain poorly understood^25,26^. Accordingly, strain injuries of the BF_lh_ remain endemic^27^ or even continue to increase^28^. Surprisingly, however, the influence of methodological differences of current eccentric hamstring strength testing on this trend has never been appropriately analysed.

It is well accepted that to establish the endurance performance of cyclists testing should be performed on a cycle ergometer and vice versa of runners on a treadmill^29^. Muscles^30^ and connective tissues also adapt to suit the task at hand^31,32^, but research only recently started to develop testing devices to measure eccentric knee flexor strength more tailored to functional requirements^33^. Yet, holistic exercise-specific evaluations are still lacking due to few valid options to reproduce standardised, controlled maximal knee flexor assessments. Thus, the appraisal of the exercise-related changes of knee flexion strength remains independent of the cohort of interest or the exercise selected related to single laboratory approaches. Practitioners and researchers use isokinetic dynamometer (IKD) data from specific settings^14,18–21,34,35^, or in exceptional cases data from a Nordic hamstring device (NHD)^6,36,37^ to obtain exact values of eccentric torque or eccentric force. Although these tests are reliable to determine eccentric knee flexor strength, a recent experiment has called them into question, indicating that hamstring muscles might be too complex to be amenable to such single test assessment^38^.

Intra- and inter-device differences of eccentric knee flexor strength might, therefore, vary to some extent to a different exercise and test similarity but fail to explain strength differences and the low within-subject correlation (r^2^ = 0.12) among professional football players. Thus, athletes may achieve similar, double, or half of strength levels on a test device compared to another device^39^. Based on these observations, we have recently conducted a comprehensive comparison study under matching biomechanical conditions of IKD and NHD tests. However, despite adjustments made to improve testing comparability between knee flexor strength tests, the correlation remained low (r^2^ = 0.34), and differences in eccentric torque outputs (~ 28%) confirmed that current test devices measure different traits^38^. This leads us to the hypothesis that training interventions based on this modality induce distinct muscle adaptations, and testing adaptations obtained with either device would give a different outcome depending on testing specificity. Therefore, a thorough comparison of these current test procedures is vital to assess the potential risk to miss a genuine intervention effect or to make overly strong claims based on random matches between the exercise and test conditions.

The purpose of this counterbalanced, randomised, single-blinded controlled trial was to elucidate the maximum possible effect of exercise and test similarity or lack thereof. Subjects were randomly assigned to two training groups, with training load reflecting precisely one or the other test evaluation. Morphological muscle responses to short-term resistive training interventions on the NHD or IKD will be assessed to clarify whether they explain potential training task specificity. We hypothesised that the exercises on the NHD and IKD stimulate significant strength gains and that intervention effects are similarly detectable by either test tool. However, owing to the increased torque with long muscle length in NHD exercise^38^, we assumed a higher increase in BF_lh_ fascicle length after NHD training than after IKD training.

## Methods

### Participants

Thirty recreationally active male sport-students from the University of Salzburg were verbally contacted to participate in this study. Twenty-five of them were previously recruited for a companion paper published elsewhere^38^. Athletic activity included football (n = 23), ice-hockey (n = 3), road cycling (n = 1), ski jumping (n = 1), alpine skiing (n = 1). Exclusion criteria were any regular lower limb resistance training, anterior cruciate ligament or other traumatic knee injuries, a history of hamstring strain, or musculoskeletal, cardiovascular, or neuronal disorders contraindicated to perform eccentric resistance training. All participants were informed regarding nature, methods, and risks associated with the experimental procedure. The research protocol was approved by the Local Research Ethics Committee (Ethikkomission, Ethikausschuss der Paris Lodron-Universität Salzburg, Kapitelgasse 4, A-5020 Salzburg; reference number: EK-GZ: 12/2017), and experiments were performed in accordance with relevant guidelines and regulations. All subjects freely gave written informed consent prior to participation. An informed consent was obtained to publish Fig. 4 of the Supplementary Dataset.

### Experimental design

A three-groups, randomised, and controlled intervention design was conducted to assess the effects of 6-week progressive hamstring exercises on an IKD (IsoMed 2000 D&R Ferstl GmbH, Hemau, Germany) or a recently validated NHD^38^.Participants were well acquainted with all test and training conditions before being assigned by simple randomisation to an isokinetic (IG), Nordic hamstring (NG), or non-exercising control group (CG). Experimental procedures involved site-specific measurements of BF_lh_ muscle size and architecture of the dominant leg^40^. Subsequently, eccentric hamstring strength was assessed in a counterbalanced design on the IKD and NHD under matched hip position and test mode speed, but retaining other inherent test modalities^38^. Accordingly, pre and post-tests were performed bilaterally on the NHD but unilaterally on the IKD in a priori block-randomised order. The participants were prohibited from taking stimulants or depressants (e.g., caffeine, alcohol) or conducting intense physical activity (e.g., running, jumping) for 6 and 24 h before all testings, respectively. All exercise interventions were supervised by the same experimenters (H-P.W, S.M, C.G), participants trained both legs, and at least one non-consecutive session per week was stored digitally. Perceived muscle soreness of the posterior thigh was recorded by filling out a line continuum of a visual analogue scale (VAS) pain score at the beginning of every session^41^. Post-tests were conducted three days after the last training session at a similar time of the day (± 2 h) as the pre-test. Participants of the CG retained their habitual daily activities but were not allowed to conduct lower limb strength training.

### Muscle strength testing

The eccentric hamstring strength tests on the IKD and NHD were preceded by a standardised warm-up on a stationary cycling ergometer (10-min, ~ 1.5 W·kg^−1^, ~ 70 rpm; Heinz Kettler GmbH and Co. KG, Ense-Parsit, Germany) and two test-like trials at ~ 80% of subjectively perceived maximum effort. Resting periods lasted for one minute between attempts of the same test and two minutes between tests. Investigators (H-P.W) provided standardised, strong verbal encouragement throughout each repetition.

**Figure 1:**
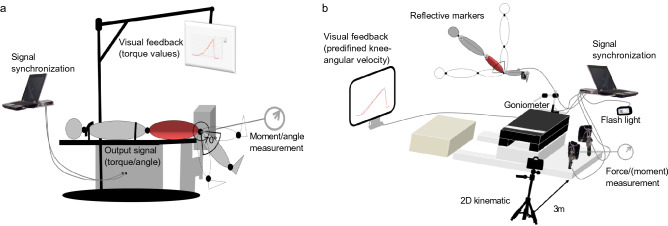
Measurement setups of the isokinetic dynamometer (**a**) and the Nordic hamstring device (**b**). The IsoMed 2000 dynamometer was calibrated according to the manufacturer's specification, and the device –integrated software saved individual settings determined in the first session. Similarly, the NHD was calibrated before the pre-tests and post-tests using standardised weights.

In IKD testing, participants lay in a supine position, with the hip joint angle set at 0° (0° = full extension) and non-tested body segments firmly fixed at anatomical positions^42^. The dynamometer axis of rotation and the knee joint centre were carefully aligned, and participants were instructed to pull the lever arm as hard and fast as possible toward the buttocks. Maximum eccentric knee flexor strength was obtained through three afterloaded isokinetic knee extensions. Hence, the dynamometers’ lever arm started a − 30° s^−1^upward movement (70–0°, 0° = full extension) when exceeding a threshold torque of 20 N·m (Fig. 1a). This preset range of motion roughly corresponds to the angles of peak torque achieved on the NHD within this population^38^.

In the NHD testing, eccentric hamstring muscle strength was determined at a predefined knee angular velocity of − 30°∙s^−1^ (NHD_30_), followed by another trial of the slowest possible knee angular velocity (NHD_max_). The participants were fixed with ankle braces at the level of the lateral malleoli so that the uniaxial load cells (Megatron Elektronik GmbH & Co. KG) assessed the forces perpendicular to the participant’s shanks. A goniometer (Biovision, Wehrheim, Germany), firmly attached to the NHD, was used to provide continuous instantaneous visual feedback during lean forward from the upright starting position (90° knee flexion) to knee joint angles of maximum eccentric knee flexor moments. Moreover, a video camera (JVC GC-PX100BEU at 50 Hz) was positioned perpendicular to the sagittal plane of motion, to capture the 2D trajectories of reflective markers attached to the participants' belly of the deltoideus muscle, trochanter major of the femur, femoral epicondyle and lateral malleolus (Fig. 1b). Nordic hamstring trials were repeated if participants were unable to match the − 30°∙s^−1^ forward lean velocity or flexed their hips (from visual inspection). A maximum of one additional repetition was required. To ensure an almost purely eccentric hamstring load, the participants bent their hips and used their arms to return to the starting position.

Dynamometer, NHD, and goniometric measurements were recorded at a 2 kHz analogue–digital conversion rate (Biovision, Wehrheim, Germany) and processed offline using a custom Matlab code (version R2017b; The MathWorks Inc., Natick, Massachusetts, USA). The 2D kinetics of the digitised reflective markers (semi-automatic video analysis; Tracker 4.87, physlets.org/tracker/) of the NHD measures were synchronised by using an electrical pulse and a flashlight. Knee joint kinetics were offset and smoothed using a digital second-order, zero-lag Butterworth filter with a cut-off frequency of 15 Hz. Torque measures on the dynamometer were corrected for gravitational and stretch-induced effects^38^. Bilateral force observations on the NHD were converted to knee joint moment by accounting for the lower-leg lever, defined as the shortest distance between the knee joint axis and the ankle strap. Out of two separate trials, the trial, including the highest eccentric torque (IKD) or the highest sum of bilateral peak forces (NHD), was retained for further analysis. Nordic hamstring tests in which the hip flexion exceeded 20° (0° = full extension) at a time or NHD_30_ trials with a mean forward lean velocity outside of − 20°∙s^−1^ to − 40°∙s^−1^ were disregarded^38^. Subsequently, all isokinetic and NHE torque values were normalised to body mass (assuming isometric scaling between torque and body mass)^4^. The average negative eccentric joint work was calculated offline as the integral of the knee joint moment over time using the trapezoidal numerical integration function^38^. Additionally, the lower limb length was determined as the shortest distance between the outermost tip of the lateral malleolus and the femoral epicondyle by using a ruler and ultrasound images.

### Training intervention

The warm-up and execution procedures on the IKD and NHD reflected the abovementioned conditions for muscle strength tests. The 6-week training volume (sets x repetitions x intensity) consisted of 2 sets of 5 repetitions, 2 days per week for the first two weeks, and then 3 sets of 5 repetitions, 3 days per week from the third week onwards. Maintaining the same number of sets and contractions has been considered the best compromise due to a lack of proper comparison of training intensity between test devices^38^. Accordingly, it remains uncertain whether the peak stress, which has been estimated to be ~ 28% higher on the NHD compared to the IKD or average work, which has been expected to be ~ 23% lower on the NHD in this cohort is the more critical mechanotransduction stimuli for morphological or performance-related hamstring muscle response^38^. Nordic hamstring exercise was performed at the NHD_30_ condition to exclude possible effects of test mode speed. The relaxing time was 3 min between sets, 15 s between repetitions, while a minimum of 24 h separated sessions. Each repetition was supervised and performed with maximum effort. Successful completion of the training regimen required the execution of at least 15 out of the 16 sessions.

### Muscle anatomical cross-sectional area and architecture

The anatomical cross-sectional area (_a_CSA), thickness and architecture of the BF_lh_ were derived using real-time two-dimensional B-mode ultrasonography (LA523, 10- to 15-MHz linear array transducer, 50 mm length, time-gain compensation at neutral position, MyLab25, Esaote, Genoa, Italy) after a five-minute rest phase^43^ while lying prone with extended hips and knees. The scan gain and depth were adjusted for each participant but held constant over the remainder of the experiment. Participants were asked to remain relaxed throughout image acquisition, and the probe was manually guided to exert low pressure on the skin but to allow finely controlled adjustments of the transducer tilt for visible muscle fascicles and almost parallel adjacent intermediate and superficial aponeurosis. Single scans were taken from the mid-distance of the medial and lateral muscle border at 25% (_a_CSA, thickness), 50% (fascicle length, pennation angle, thickness), and 75% (thickness) of the BF_lh_. Positions were manually determined from a distance between the ischial tuberosity and the distal lateral femoral condyles. Anatomical landmarks were traced on a transparent acetate paper and used to ensure that subsequent scanning measurements were taken at the same site^44^. All scans of the BF_lh_ were conducted by the same experienced sonographer (H-P.W.), and analysed offline within a few consecutive days, blinded to the participants’, using the image-processing program Fiji (ImageJ, 1.51n, Rasband, W.S., National Institutes of Health, Bethesda, MD, USA). Therein BF_lh a_CSA was segmented manually with a polygon selection tool, and fascicle length was obtained through a linear extrapolation as described previously^32,45,46^. The pennation angle was defined as the angle of the fascicle relative to the deeper aponeurosis. The BF_lh_ muscle thickness at 50% and 75% were measured as a surrogate of _a_CSA. The validity^47^ and reliability^48^ of this method have been shown previously (Fig. 1a–d in Supplementary Dataset).

### Statistics

**Figure 2:**
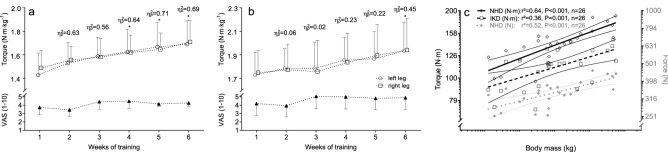
Time course of changes in body mass normalised eccentric knee flexor torque and muscle soreness measures on the dynamometer (IKD) (**a**) and Nordic hamstring device (NHD) (**b**) and body mass plotted against torque (IKD) and force (NHD) values (**c**). Average weekly soreness measured using a numeric visual analogue scale (VAS) (1–10) at the beginning of each training session. Values are mean ± 95%CI. * p < 0.05 (Bonferroni-corrected) versus the average torque of the first intervention week. Data points of body mass were obtained from the baseline characteristics of the recruited subjects. The linear regressions and their 95% confidence limits indicate that body-mass-correlate with the peak eccentric torque (IKD) and peak eccentric force (NHD).

G*Power (G*Power®, Version 3.1.9.2.) was used to calculate the sample size necessary to achieve moderate to large changes in EPT, as outlined in comparable studies (6-week progressive eccentric IKD training, Cohen’s d range 0.63–0.78^14^ or 10-week progressive eccentric NHD training, Cohen’s d range 1.55–2.36^13,37^). Conservative a priori power analysis (Cohen’s d = 0.63, power = 0.80, alpha = 0.05, correlation among repeated measures = 0.5) revealed a group size of 9 participants. Thirty participants were recruited in anticipation of a dropout rate of one participant per group. Raw data or sampling distribution of the change score were evaluated visually and statistically (Shapiro–Wilk test) for normality of distribution. Baseline differences were tested for each variable with a one-way independent analysis of variance (ANOVA). Bonferroni-adjusted simple contrasts analyses were used to determine the time course of average knee flexor torque and muscle soreness (VAS) developments during the six-week intervention phase. The effects of the intervention on each variable were determined with a three-way mixed factorial ANOVA [3 (groups) × 2 (times) × 2 (devices)]. Within each test device and in case of significant interaction effects, a one-way independent ANOVA was used to compare post-to-pre variable differences between each group and Tukey-adjusted P-values to evaluate which group(s) differed over time. Per cent changes are described as changes in the intervention group by subtracting the control group's changes. The effect size eta squared (η^2^_p_) was deemed small (η^2^_p_ > 0.01), medium (η^2^_p_ > 0.06), or large (η^2^_p_ > 0.14)^49^. F- or Q-statistics and P-values are shown so that the relative degree of significance can be assessed in all cases. Individual responses to the treatment were summarised by standardising the variation in change scores of the experimental groups added to the variation arising from the error of biological plus instrumentation noise^50,51^. We assumed similar error of measurements within the experimental and control groups since participants were well habituated to all test conditions, and no effect for time has been found on the eccentric knee flexor strength for either device^38^. The smallest important effect was considered as 0.2 of the between-subject standard deviation (4%). The threshold for interpreting standardised mean changes were 0.2, 0.6, 1.2, 3.0, and 4.0 for small, moderate, large, very large, and extremely large. The same thresholds halved were used for standardised standard deviations^50^. The 90% confidence limits for probabilities of responders were derived using a bootstrap method. Pearson’s correlation coefficients were used to determine the relationships between eccentric strength and participants’ body mass. All statistical analyses were conducted using SPSS Statistics V.26.0 (IBM Corporation, Chicago, Illinois, USA) or adopted Excel-Templates^51^. Figure 1 was created by H-P.W, using laboratory photos and drawings in Microsoft Office Excel Professional Plus 2016 (Redmond, WA, USA). Figure 2, 3 were created using the GraphPad Prism 9.0.0 (GraphPad Software, La Jolla, CA, USA, https://www.graphpad.com/scientific-software/prism/). Unless otherwise stated, data are reported as means and standard deviations (SD), and the level of significance was set at P = 0.05.

**Figure 3:**
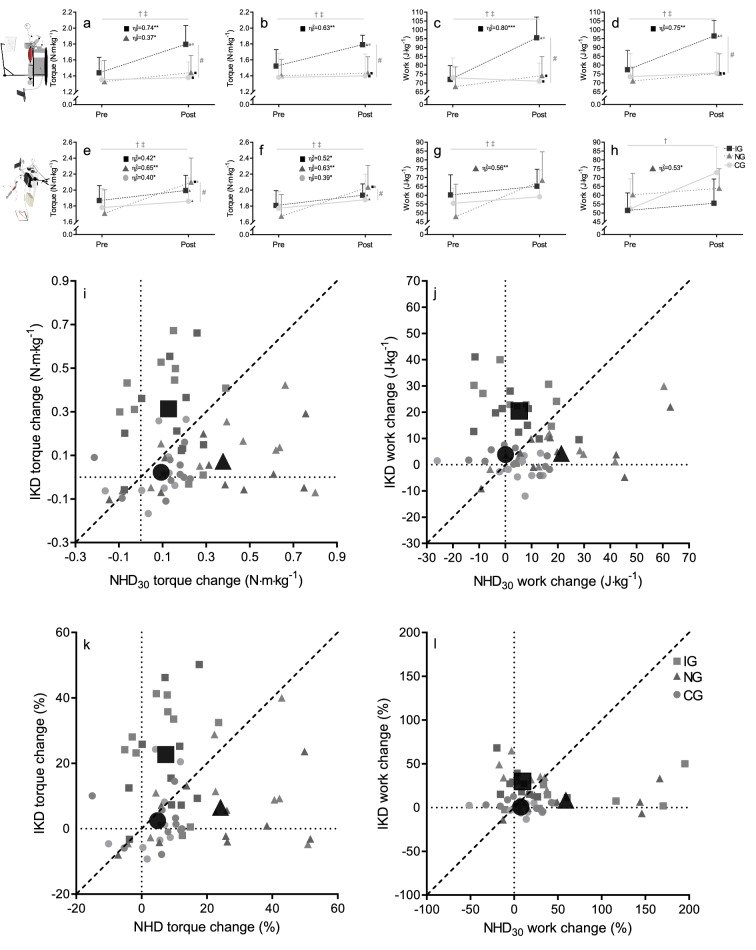
Pre- and post-performance in eccentric torque and work measured with the isokinetic device (left: **a**,**c**; right: **b**,**d**) and the Nordic hamstring device (left: **e**,**g**; right: **f**,**h**) and the relation of obtained torque (**i**,**k**) and work (**j**,**l**) changes over time. Values are mean ± SD. IG, isometric group; NG, Nordic hamstring group; CG, control group; IKD, isokinetic device; NHD_30_, Nordic hamstring test at a lean forward velocity of ~ 30°·s-1. # for the significant group effect, with post-hoc differences between groups shown by squares (significantly different from IG), triangles (significantly different from NG), and circles (significantly different from CG); † for the significant time effect; ‡ significant interaction effect. Further, each subgroup was analysed independently (paired sample t-test); *, P < .05, **, P < .01, ***, P < .001.

## Results

All participants completed the designed experiment (training compliance rate: IG: 96.3 ± 3.2%; NG: 95.6 ± 3.0%), and the maximal (IG) and supramaximal (NG) eccentric training loads triggered low muscle soreness (average 6-week VAS score: IG: 4.2 ± 1.5 vs NG: 4.7 ± 2.1; Fig. 2a,b, Fig. 2a,b in Supplementary Dataset). In NHD training and testing, participants could not resist in any repetition the body weight-induced moment until full knee extension (NHD_30_ and NHD_max_, angle of peak torque ≥ 15.2°; 0° = full extension). Correlation analyses indicated a highly significant relationship between eccentric strength measurements on either device and participants’ body mass (Fig. 2c). However, physical characteristics, including strength modifiers like lower-leg lever length or body mass, yielded no differences between groups (Table 1) and therefore only influenced the interpretation to a small extent. Besides, it has been shown that NHD_max_ and NHD_30_ measures of EPT and work can be used interchangeably^38^, and body mass corrected performance changes after the six-week intervention period did not differ between groups when measured with both approaches (P ≥ 0.256, η^2^_p_ = 0.05). Accordingly, all raw values and NHD_max_ measures remain tabulated and presented in the Supplementary Dataset but are only discussed when appropriate.

**Table 1:** Physical characteristics of participants.

	Isokinetic	Nordic Hamstring	Control	F_(2;27)_	P-Value	η^2^_p_-Value
Sample size (n)	10	10	10			
Age (yrs)	25.9 ± 2.6	25.0 ± 2.9	26.2 ± 2.3	0.57	0.575	0.04
Body height (cm)	181.5 ± 8.3	181.4 ± 5.1	180.6 ± 5.5	0.05	0.948	< 0.01
Body mass (kg)	80.4 ± 10.7	80.8 ± 10.6	78.7 ± 7.5	0.14	0.872	0.01
BMI (kg∙m^2–1^)	24.3 ± 2.0	24.5 ± 2.6	24.1 ± 2.0	0.08	0.922	< 0.01
Lower limb length left (cm)	42.1 ± 1.9	43.4 ± 2.2	42.5 ± 2.5	0.31	0.737	0.02
Lower limb length right (cm)	42.0 ± 2.0	43.3 ± 2.5	42.7 ± 2.2	0.12	0.886	< 0.01

Values are express as mean SD; BMI, body mass index.

### Eccentric muscle torque and work

The term “normalised” is hereinafter used in combination with EPT or work instead of “normalised to body mass”. At baseline, normalised eccentric peak torque and normalised average work did not differ between groups (IKD: F_(2;27)_ ≤ 0.98, P ≥ 0.389, η^2^_p_ ≤ 0.07; NHE_30_: F_(2;27)_ ≤ 1.08, P ≥ 0.352, η^2^_p_ ≤ 0.07). However, normalised eccentric peak torque was generally higher when measured on the NHD_30_ than the IKD (left: + 30%, t = 8.63, P < 0.001, η^2^_p_ = 0.72; right: + 22%, t = 5.99, P < 0.001, η^2^_p_ = 0.55). In contrast, normalised total eccentric work was lower in NHD_30_ than IKD measures (left: -29%, t = 5.56, P < 0.001, η^2^_p_ = 0.52; right: -26%, t = 6.27, P < 0.001, η^2^_p_ = 0.58).

The time course of the average unilateral normalised torque of each training session is presented in Fig. 2a,b. Exercise on the IKD revealed a significant main effect of time (F_(1;5)_ = 15.22, P < 0.001, η^2^_p_ = 0.63), which was primarily driven after the third week of training. Similarly, the mean normalised torque output on the NHD increased over time (main effect: F_(1;5)_ = 3.89, P = 0.005, η^2^_p_ = 0.30), with a significant increase in the sixth week only (P = 0.024). There was no effect of leg (main effect: IKD: F_(1;5)_ = 0.11, P < 0.745, η^2^_p_ = 0.01; NHD: F_(1;5)_ < 0.01, P < 0.994, η^2^_p_ < 0.01). The VAS scores only provided a significant main effect of time on exercises on the NHD (IKD: F_(1;5)_ = 1.81, P = 0.131, η^2^_p_ = 0.17; NHD: F_(1;5)_ = 2.87, P = 0.025, η^2^_p_ = 0.24), but simple contrast analyses in the IG and NG yielded no significant effects in any week compared to baseline.

In normalised EPT, significant group × time × device effects (left: F_(2;27)_ = 10.68, P < 0.001, η^2^_p_ = 0.44; right: F_(2;27)_ = 11.53, P < 0.001, η^2^_p_ = 0.46) and highly significant main effects for devices (left: F_(2;27)_ = 109.08, P < 0.001, η^2^_p_ = 0.80; right: F_(2;27)_ = 60.11, P < 0.001, η^2^_p_ = 0.69) were found. Similar, in normalised eccentric work, significant group × time × device effects (left: F_(2;27)_ = 12.68, P < 0.001, η^2^_p_ = 0.48; right: F_(2;27)_ = 8.03, P = 0.002, η^2^_p_ = 0.37) and highly significant main effects for devices (left: F_(2;27)_ = 39.86, P < 0.001, η^2^_p_ = 0.60; right: F_(2;27)_ = 53.08, P < 0.001, η^2^_p_ = 0.66) were discovered.

On the IKD, there were significant group × time interactions for the normalised EPT and normalised average work (Table 2). Changes in normalised EPT were significantly higher in the IG compared to the CG (left: + 23%, Q_(2,18)_ = 4.19, P = 0.001, η^2^_p_ = 0.47; right: + 16%, Q_(2,18)_ = 3.70, P = 0.003, η^2^_p_ = 0.40) and compared to the NG (left: + 17%, Q_(2,18)_ = 3.11, P = 0.012, η^2^_p_ = 0.31; right: + 16%, Q_(2,18)_ = 3.46, P = 0.005, η^2^_p_ = 0.33). There was no significant difference observed in normalised EPT between other groups (Fig. 3a,b). Similar, changes in normalised eccentric work were significantly higher in the IG compared to the CG (left: + 35%, Q_(2,18)_ = 6.55, P < 0.001, η^2^_p_ = 0.73; right: + 19%, Q_(2,18)_ = 4.90, P < 0.001, η^2^_p_ = 0.60) and compared to the NG (left: + 24%, Q_(2,18)_ = 4.48, P < 0.001, η^2^_p_ = 0.45; right: + 19%, Q_(2,18)_ = 4.21, P = 0.001, η^2^_p_ = 0.42). No other group differences were found for normalised EPT and normalised average work (Fig. 3c,d).

**Table 2:** Eccentric peak torque and work obtained during the isokinetic and Nordic hamstring exercise tests.

		Isokinetic		Nordic Hamstring		Control		Interaction (group × time		
		Pre-Test	Δ(post–pre)	Pre-Test	Δ(post–pre)	Pre-Test	Δ(post–pre)	F_(2;27)_	P-Value	η^2^_p_-Value
IKD (unilateral—left leg)	EPT (N∙m)	115.7 ± 21.7	28.2 ± 18.6	107.4 ± 25.7	8.4 ± 11.6	106.1 ± 19.2	2.3 ± 10.8	9.18	0.001	0.41
	EPT (N∙m∙kg^−1^)	1.44 ± 0.19	0.36 ± 0.22	1.33 ± 0.27	0.11 ± 0.15	1.35 ± 0.24	0.03 ± 0.14	9.48	0.001	0.41
	Average work (kJ)	5.79 ± 0.98	1.88 ± 0.97	5.48 ± 1.07	0.45 ± 0.66	5.73 ± 0.97	− 0.14 ± 0.35	21.27	< 0.001	0.61
	Average work (J∙kg^−1^)	72.01 ± 7.82	23.52 ± 10.51	67.99 ± 11.09	6.17 ± 9.57	72.92 ± 11.22	− 1.88 ± 4.82	22.44	< 0.001	0.62
IKD (unilateral—right leg)	EPT (N∙m)	122.0 ± 20.5	21.5 ± 17.3	113.6 ± 23.8	2.5 ± 9.7	108.5 ± 24.9	1.0 ± 6.6	8.98	0.001	0.40
	EPT (N∙m∙kg^−1^)	1.52 ± 0.21	0.27 ± 0.22	1.40 ± 0.21	0.03 ± 0.13	1.38 ± 0.30	0.02 ± 0.8	8.59	0.001	0.39
	Average work (kJ)	6.20 ± 1.07	1.55 ± 0.94	5.73 ± 0.90	0.32 ± 0.66	5.78 ± 1.10	0.14 ± 0.28	12.75	< 0.001	0.49
	Average work (J∙kg^−1^)	77.44 ± 10.99	19.10 ± 9.74	71.10 ± 7.78	4.35 ± 8.64	73.54 ± 13.06	4.35 ± 8.64	14.07	< 0.001	0.51
NHD_**30**_ (unilateral—left (30°s))	EPT (N∙m)	150.6 ± 27.8	10.0 ± 12.5	139.0 ± 35.0	31.7 ± 25.8	140.0 ± 23.5	6.5 ± 8.3	6.30	0.006	0.32
	EPT (N∙m∙kg^−1^)	1.87 ± 0.19	0.13 ± 0.16	1.71 ± 0,30	0.39 ± 0.30	1.78 ± 0.24	0.08 ± 0.10	6.79	0.004	0.34
	Average work (kJ)	4.86 ± 1.17	0.40 ± 0.82	3.99 ± 1.85	1.50 ± 1.40	4.37 ± 0.84	0.31 ± 0.89	3.82	0.035	0.22
	Average work (J∙kg^−1^)	60.21 ± 11.38	4.92 ± 10.75	47.98 ± 18.25	20.49 ± 20.17	55.51 ± 8.51	3.63 ± 11.57	4.03	0.029	0.23
NHD_**30**_ (unilateral—right (30°/s))	EPT (N∙m)	145.6 ± 25.5	10.5 ± 10.3	136.5 ± 35.4	28.3 ± 23.3	139.1 ± 20.0	8.5 ± 10.8	4.66	0.018	0.26
	EPT (N∙m∙kg^−1^)	1.81 ± 0.18	0.13 ± 0.13	1.67 ± 0.27	0.36 ± 0.29	1.77 ± 0.21	0.11 ± 0.14	4.88	0.016	0.27
	Average work (kJ)	4.83 ± 1.06	0.27 ± 0.97	4.34 ± 1.82	20.46 ± 22.69	4.03 ± 0.79	0.36 ± 0.91	3.23	0.055	0.19
	Average work (J∙kg^−1^)	60.29 ± 12.07	3.63 ± 12.00	52.34 ± 16.97	1.50 ± 1.61	51.47 ± 9.89	4.04 ± 11.00	3.55	0.043	0.21
	Mean angular velocity (°·s^−1^)	26.7 ± 3.7	− 0.2 ± 6.0	30.3 ± 4.7	− 5.3 ± 5.8	26.7 ± 4.8	3.6 ± 4.1	6.90	0.004	0.34
NHD_**max**_ (unilateral—left (max))	EPT (N∙m)	151.7 ± 28.0	6.9 ± 8.7	141.9 ± 40.1	26.8 ± 24.4	142.1 ± 26.9	2.2 ± 11.5	6.33	0.006	0.32
	EPT (N∙m∙kg^−1^)	1.88 ± 0.22	0.09 ± 0.20	1.74 ± 0.34	0.35 ± 0.31	1.80 ± 0.30	0.03 ± 0.15	6.51	0.005	0.33
	Average work (kJ)	4.92 ± 1.21	0.74 ± 1.17	4.22 ± 1.86	0.97 ± 1.75	4.70 ± 1.07	− 0.32 ± 1.10	2.55	0.097	0.16
	Average work (J∙kg^−1^)	61.08 ± 11.99	9.28 ± 14.06	51.50 ± 19.87	13.15 ± 21.49	60.12 ± 14.71	− 4.70 ± 14.21	3.07	0.063	0.19
NHD_**max**_ (unilateral—right (max))	EPT (N∙m)	150.6 ± 26.7	7.7 ± 17.3	145.4 ± 38.4	18.1 ± 23.2	144.8 ± 23.8	1.7 ± 9.7	2.22	0.128	0.14
	EPT (N∙m∙kg^−1^)	1.87 ± 0.19	0.10 ± 0.20	1.79 ± 0.35	0.23 ± 0.29	1.84 ± 0.28	0.02 ± 0.12	2.60	0.093	0.16
	Average work (kJ)	4.81 ± 1.10	0.48 ± 0.56	4.35 ± 1.52	1.36 ± 1.53	4.68 ± 1.17	− 0.50 ± 1.27	6.04	0.007	0.31
	Average work (J∙kg^−1^)	59.89 ± 13.03	6.20 ± 7.59	53.01 ± 15.63	17.99 ± 20.91	59.79 ± 15.42	− 6.88 ± 16.12	6.16	0.006	0.31
	Mean angular velocity (°·s^−1^)	11.3 ± 2.9	0.2 ± 2.9	13.2 ± 5.9	0.1 ± 6.1	15.3 ± 6.5	-2.8 ± 3.6	1.47	0.249	0.10

Values are express as mean SD; IKD, isokinetic device; NHD30, Nordic hamstring test at a lean forward velocity of ~ 30° s − 1; NHDmax, Nordic hamstring test at low lean forward velocity; EPT, eccentric peak torque.

On the NHD, there were significant group × time interactions for the normalised EPT (Table 2). Post-hoc analysis indicated that changes in normalised EPT were higher in the NG compared to the CG (left: + 18%, Q_(2,18)_ = 3.42, P = 0.006, η^2^_p_ = 0.38; right: + 15%, Q_(2,18)_ = 2.81, P = 0.024, η^2^_p_ = 0.25) and compared to the IG (left: + 15%, Q_(2,18)_ = 2.90, P = 0.019, η^2^_p_ = 0.25; right: + 14%, Q_(2,18)_ = 2.58, P = 0.040, η^2^_p_ = 0.23). There was no significant difference observed in normalised EPT between other groups (Fig. 3e,f). Despite some significant group × time interactions for the average normalised work, the changes in work produced during the NHE_30_ was only higher in the NG compared to controls of the left leg (+ 35%, Q_(2,18)_ = 2.55, P = 0.043, η^2^_p_ = 0.35) (Fig. 3g,h).

There was a significant group × time interaction for the mean lean forward velocity of the NHD_30_ trials, with a higher increase in mean lean forward angular velocity in the CG compared to the slight decrease in mean angular velocity of the NG (Q_(2,18)_ = 3.70, P = 0.003, η^2^_p_ = 0.47). Within-group comparisons yielded a lower lean forward velocity in the post-test of the NG (t = -2.88, P = 0.018, η^2^_p_ = 0.48), no change in the IG (t = -0.08, P = 0.938, η^2^_p_ = 0.07), while the mean angular velocity of the CG increased (t = 2.80, P = 0.021, η^2^_p_ = 0.46) (Table 2).

### Muscle anatomical CSA and architecture

There was no baseline difference (F_(2;27)_ ≤ 1.98, P ≥ 0.157, η^2^_p_ ≤ 0.13) and no significant group × time interaction, as well as no main effect for anatomical and architectural features of the BF_lh_ (Table 3).

**Table 3:** Biceps femoris long head morphology parameters and pre- and post-test.

	Isokinetic		Nordic hamstring		Control		Interaction (group × time		
	Pre-test	Δ(post–pre)	Pre-test	Δ(post–pre)	Pre-test	Δ(post–pre)	F_(2;27)_	P-Value	η^2^_p_-Value
aCSA 25% (cm^2^)	6.4 ± 1.9	0.2 ± 0.5	6.1 ± 1.9	0.3 ± 0.4	4.5 ± 1.7	0.1 ± 0.7	0.225	0.800	0.016
MT 50% (mm)	33.2 ± 6.2	0.2 ± 0.8	30.0 ± 5.0	1.2 ± 1.7	28.4 ± 4.8	0.2 ± 1.1	1.886	0.171	0.123
MT 75% (mm)	28.5 ± 5.3	0.4 ± 0.8	27.7 ± 4.5	− 0.1 ± 1.7	29.0 ± 2.8	0.2 ± 1.3	0.285	0.754	0.021
PA 50% (°)	11.8 ± 2.6	− 0.2 ± 1.1	12.2 ± 1.4	− 0.7 ± 1.6	11.8 ± 1.5	0.1 ± 1.4	2.036	0.150	0.131
BF_lh_ FL 50% (mm)	117.4 ± 26.1	0.5 ± 0.7	118.1 ± 15.2	− 0.1 ± 1.3	110.4 ± 19.9	0.4 ± 1.3	0.820	0.451	0.057

MT, muscle thickness; aCSA, anatomical cross-sectional area; PA, pennation angle; BF_lh_, biceps femoris long head; FL, fascicle length.

### Individual responses

Pre-to-post change scores in muscle torque (IKD: t = 1.74, P = 0.093, η^2^_p_ = 0.09; NHD: t = 0.03, P = 0.976, η^2^_p_ = 0.01) and work (IKD: t = 0.53, P = 0.598, η^2^_p_ = 0.01; NHD: t = 0.21, P = 0.976, η^2^_p_ < 0.01) were similar between the left and right leg (Fig. 3a-h) but provided a high intraindividual variability on both devices (Fig. 3i-l).

Demonstrated for the strength measurements, the overall standardised effect of isokinetic training on the IKD ranged from moderate to very large when measured on the IKD (mean ± SD; left: = 0.81 ± 2.29 N·m·kg^−1^; right: 0.62 ± 2.34 N·m·kg^−1^), when the effect of noise in the sample mean and SD was eliminated. However, when measured on the NHD, moderate negative to large positive changes are reasonably compatible with our observed data (left: = 0.21 ± 0.77; right: = -0.28 ± 0.56). The overall effect of isokinetic training on the NHD_30_ on individuals ranged typically from borderline small to moderate when measured on the IKD (left: = 0.05 ± 0.99; right: mean ± SD = 0.32 ± 0.82), but moderate to very large when measured on the NHD (left: = 0.45 ± 2.75; right: = 0.47 ± 2.75) (Table 4).

**Table 4:** Estimation of individual response to isokinetic and Nordic hamstring exercise interventions.

	Isokinetic device				Nordic hamstring device			
	Isokinetic		Nordic Hamstring		Isokinetic		Nordic Hamstring	
	Left leg	Right leg	Left leg	Right leg	Left leg	Right leg	Left leg	Right leg
SWC (%)	3.37	3.30	3.37	3.30	2.75	2.58	2.75	2.58
Mean change (N∙m∙kg^−1^)	0.36	0.34	0.11	0.03	0.13	0.13	0.39	0.36
SD_IR_ (N∙m∙kg^−1^)	0.17	0.20	0.06	0.10	0.12	− 0.06	0.28	0.26
Proportion of response (%; [CL])	96.6 [83–100]	93.2 [86–100]	85.1 [51–99]	44.6 [15–72]	74.7 [47–98]	93.0 [58–100]	88.8 [75–99]	89.2 [75–99]
Standardised mean change (a.u.)	1.55	1.48	0.49	0.14	0.52	0.57	1.60	1.61
Standardised SD_IR_ (a.u.)	0.74	0.86	0.28	0.42	0.47	− 0.25	1.15	1.14

SCW, smallest worth change; SDIR, standard deviation of individual response; CL, confidence limits of proportion of response (%).

## Discussion

The most crucial findings of the present study are the enormous exercise specificity of eccentric knee flexor training and the testing specific evaluations of their efficiency and effectiveness. Accordingly, in the present study meaningful intervention effects were only observed when changes in knee flexor torque or work were derived from the training device. This insensitivity of conventional knee flexor testing to experiment-specific variations of muscle loading harbours the inherent risk of missing genuine and potentially meaningful training effects (inflating Type II error rate) or drawing inappropriate causal inferences concerning injury prevention or rehabilitation (Type I error). Methodological test differences are, therefore, likely to contribute to current gaps in classifying effective but efficient hamstring exercises, urgently needed in terms of athletic performance and muscle tissue integrity. Contrary to our hypothesis, observed changes in knee flexor strength were not reflected in site-specific morphological and architectural muscle parameters when measured at rest. These observations suggest that the specificity of short-term training effects can also be primarily related to neural rather than structural adjustments.

### Eccentric muscle torque and work

Despite adjustments made to the biomechanical parameters of the IKD and NHD tests, we only observed systematic changes in the knee flexor strength (Fig. 3a,b,e,f) and average work (Fig. 3c,d,g,h) under matched test and intervention modalities. This lack of reciprocal transfer effects between conventional devices contradicts our expected similarity hypothesis to detect changes in hamstring muscle strength after exercise intervention. Still, it indicates that eccentric exercises on IKD and NHD trigger highly specific muscle responses. However, the test methods decide whether these adjustments to the muscle tissue can be detected or not. Provided tests were recorded on the training device, observed increases of knee flexor strength are mostly consistent with previous scientific results.

When measured on the IKD (Fig. 3a,b), exercise-induced gains in normalised EPT of either leg of 18%-to-25% (η^2^_p_ ≥ 0.63) of the IG seem to confirm the consistent effectiveness of isokinetic eccentric exercise^12,14,18,20^. Similar, the lower effects of 2%-to-8% (η^2^_p_ ≥ 0.07) of the NG, in which a decrease of 0.04 N·m·kg^−1^ to an increase of 0.22 N·m·kg^−1^, or -3 N·m to + 16 N·m (BCa 95%, see also Table 1 of the Supplementary Dataset) is also reasonably compatible with our non-significant data, are comparable with some previous observations^15,19,52^. Other studies, however, also found higher increases in this parameter^21,34,35,53^. This inconsistent outcome of the efficacy of Nordic hamstring exercise, when measured on an IKD (range: 2%^15^ -to- 21%^35^), remains challenging to reconcile in consideration of methodological heterogeneity of training and testings. Accordingly, interventions of Nordic hamstring exercise differed between 4^34,35,52,53^ -to- 10^19^ weeks, 9^35^-to-27^19^ sessions, and in total 108^52^ -to- 736^19^ repetitions. Muscle loads were also influenced by combining Nordic hamstring exercise with other interventions^15^, manipulating forward-leaning velocities^52^, or increasing eccentric loads with additional torque beyond body mass^19^. Moreover, dynamometer tests were performed under different conditions related to the range of motion (55°^34^-to-110°^52^), isokinetic joint velocity (− 30°∙s^−1^^52^-to- − 240°∙s^−1^^35^) and hip position (90°^15^ -to- 100°^21,53^ flexed to prone position^35,52^).

When measured on the NHD (Fig. 3e,f), body mass normalised strength gains of either leg of 22%-to-23% (η^2^_p_ ≥ 0.63) were observed in the NG. This finding is consistent with previous observations and seems to confirm the consistently proven effectiveness of Nordic hamstring exercise in improving eccentric knee flexor strength (+ 15%^36^-to- + 27%^13,37^). In contrast, alterations of knee flexor strength following isokinetic exercise has not been evaluated via an NHD so far. However, similar to IKD testing, there are considerable differences in NHD measurements. Indeed, the NHD test should not be deprived of its practicability, but researchers emphasise that physical characteristics of body mass and lower limb length are associated with NHD output measures^4^. The former has been considered sporadically^15,21,52^, but individual differences in lower-leg lever length have remained unaccounted in intervention studies so far. In the present study, some of the exercising or control sport-students were heavier (range: 64 kg-to-97 kg) or taller (range: 169 cm-to-191 cm) than others, and analyses revealed a highly significant, positive relation between participant’s body weight and eccentric strength values (IKD: + 2.1 N·m·kg^−1^ (4.3 N·kg^−1^); NHD: + 1.3 N·m·kg^−1^; Fig. 2c). Using a ceteris paribus assumption but account for individual differences in lower leg lever length (present study: 39 cm-to-45.5 cm), physics reveals that taller subjects with longer lower limbs produce lower strength values than those of shorter lower limbs on NHD. In reality, individual scaling differences are subdued to complex interactions. Taller subjects can be expected to have a greater hamstring muscle moment arm with individual but unknown changes to dynamic muscle actions^54^. This indeterminacy of internal moment arms precludes certainty of muscle strength measures using conventional tests. However, while external moments such as the length of the lower leg lever do not play a role on IKD, provided the knee and dynamometer axes remain aligned, neglecting these variables entails the risk of misinterpretation load cell forces on Nordic hamstring devices.

Accordingly, ratio normalisation to body mass and lower-leg lever length differences improve the test validity of eccentric knee flexor strength assessment^4^. However, we also acknowledge that there exists no “gold standard” normalisation procedure, and interpretation problems of correlational or experimental research should be avoided by aligning relevant covariates between-groups. Hence, in present findings, ratio related eccentric knee flexion torque measures on IKD allows for comparison of subjects of different mass, but normalised NHD hamstring strength values remained elevated in heavier participants (see Fig. 2c in Supplementary Dataset). Alternatively, one could argue that the interpretation of reciprocal transfer effects between IKD and NHD measures could be biased by exercise-related dissimilarity of external angular work and peak muscle tension. Importantly, however, this would be at odds with comparable increases of relative strength (20% or slightly above), and average work (about 30%) of both experimental groups when measured on the training device. Besides, the counterbalanced design, the matching of other biomechanical parameters of IKD and NHD training and testing, the similarity of physical characteristics between groups (Table 1) or between raw and body mass normalised data (Table 2), suggest that anthropometric differences of participants unlikely influenced the present study’s between groups-comparisons. Instead, current data suggest an enormous sensitivity of hamstring muscles beyond the type of contraction, angular velocity, and a similar range of motion. The scope of these observed, mechanical stimuli related differences between the isokinetic (IKD) and isoweight (NHD) exercise on muscle strength, as well as the responsible mechanisms have to be ascertained in future studies.

### Changes in muscle anatomical CSA and architecture

Non-significant radial muscle response following weeks of eccentric training is frequently observed^55^, but the lack of longitudinal muscle response contrast previous findings^12–17^ and challenges our hypothesis (Table 3). Hence, eccentric exercises in animal studies have shown stimulation of sarcomerogenesis^56,57^, and fascicle length in human BF_lh_ increased after weeks of Nordic hamstring^13,16^ or eccentric isokinetic exercise^12,14^. Albeit hypothetical, this absent structural re-assembly within the BF_lh_ may reflect regulative mechanisms that depend on the methodological approach. The deposition of synthesised serial sarcomeres in muscle fibres is associated, inter alia, with preceded muscle damage^56^. In support of this, Brockett and colleagues^22^ have shown a shift of the moment–angle relationship towards a greater knee angle –indirect evidence of an increase in the number of sarcomeres in series—only after intervention periods of high muscle soreness. While muscle damage remained unexplored in the intervention studies mentioned above, the indirect marker of low VAS scores in the present study (Fig. 2a,b) does not indicate any profound molecular and cellular mechanisms of tissue damage and repair. Alternatively, unprovoked muscle tissue response could be ascribed by study-related differences in the exercise range of motion, lengthening velocity^58^, or hip position^12^. Accordingly, Seymore and colleagues^15^, who also controlled hip angles at 0° during the Nordic hamstring exercise, could not find any effect on muscle architecture either. Moreover, muscular chains, with bidirectional interactions between the connective tissue (aponeurosis, external tendons) and differences in muscle fibre longitudinal and radial strain, including decoupling of the fibre length trajectories from the muscle–tendon unit (e.g., tendon or muscle belly gearing), or factors like history-dependency on force generation likely also play a significant role to different neuronal and structural changes due to eccentric knee flexor training. Hence, it is also possible that the BF_lh_ fascicle strain is limited within the measured region of interest at 50% of the muscle length and that the specified (IKD) or force-determined (NHD) range of motion does not cause length changes of the sarcomeres towards the descending force–length relationship. This would again fit with the relatively stable but low VAS scores, as well as that the highest torque outputs were measured at long muscle length (~ 65°, Fig. 3a,b in Supplementary Dataset). Thus, hamstring muscle likely worked near-optimal muscle length towards the end of the exercise but provided strength gains (IKD) over nearly the whole range of motion (Fig. 3c,d in Supplementary Dataset).

Also worth mentioning, knee flexor strength improvement showed no levelling-off after the 6-week intervention period and potentially required assumptions in using B-mode ultrasonography measures in vivo (see Franchi et al.^59^ for review) caused an insensitivity to detect some genuine morphological muscle adaptation. Reducing the likelihood and effects of these errors (e.g., associated with the narrow field of view), together with an investigation over a longer training period and of other hamstring muscles will allow the present findings to be confirmed.

### Individual responses

Inherent individual mechanotransduction capabilities seem to be critical determinants of hamstring muscles, and intervention effects ranged from being entirely ineffective to substantial increases in EPT and work. Part of this variability on the NHD might be explained by variations in extrinsic manipulation, with individuals experiencing stimuli of different sizes for adaptive reactions due to differences in upper body composition. However, this variability cannot explain changes between -7% to + 51% in EPT. Even more, it fails for measures on the IKD, where a similar training dose is expected among individuals, but still, the variability of the change scores was comparably high (-8% to + 50%) (Fig. 3i–l). Although low sample size warrants caution of interpreting individual training effects, the responses as proportions of responders and the associated confidence limits indicated that even up to one-quarter of individuals do not show meaningful increases in eccentric knee flexor strength under matched test and intervention modalities. Yet, despite the matching of biomechanical parameters of IKD and NHD testing, the prevalence of “non-responders” to eccentric exercise dramatically increase when measured on the other devices. Given the observed data, evaluations of isokinetic training effects using the NHD revealed, for example, that up to 85% of individuals do not augment their strength capacity higher than the smallest worth changes plus the typical error of measurement (Table 4).

Hence, exercise specificity and test specificity and their uncontrolled combination raise compelling challenges to personalised preventive or rehabilitative medicine or performance enhancement exercise selections. We should rather accept that there will probably never exist a single muscle contraction mode (eccentric, concentric, isometric)^60^ or exercise typology (isoinertial, isokinetic or isoweight), nor a separate testing device to assess beneficial intervention effects.

## Conclusion

The present findings clearly highlighted the potential lack of sensitivity of current test devices to assess eccentric knee flexor strength gains. Thus, the judgment of exercise inclusion in prevention or rehabilitation guidelines can be, to a greater extent, determined by the similarity of the intervention exercise to the methodological strength tests rather than by contributing to reduce the risk of hamstring strain injury. Consequently, great attention should be devoted to lowering biased tests of hamstring strength assessments with real costs in health and dollars. To this end, clinicians, sports injury researchers, or other practitioners should be guided by evidence-based theory, with in-depth knowledge of the anatomical and physiological background. However, we have to recognise that the assessment of hamstring strength remains a compromise between practicability, standardizability, and proximity to real-life conditions. Single test tools will never reflect all functional requirements of the muscle–tendon complex, and there will likely ever be the best or most valid knee flexor strength assessment with an inferential capacity to all individuals' real-life situations of performance or integrity. Instead of the reductionist approaches of using either dynamometer or a Nordic hamstring device, we propose a set of different strength test options tailored to athletes or patients to identify muscular deficits. However, to implement this, there is an urgent need for further development for static, but even more so for dynamic, real-time analysis, including the detection of muscle architectural remodeling^45^. Ultimately, investigations of these mechanisms could contribute to gain insight into interindividual determinants of eccentric knee flexor strength trainability and reduce the widespread but severe hamstring injury in the long term. Future studies with extended training periods and a better imaging approach to reflect potential muscle remodeling are needed to confirm our present interpretations and the lack of changes in muscle morphological and architectural characteristics.

## Limitations

Potential limitations should be considered before concluding. First, the accuracy of BF_lh_ ultrasound measures depends on accessor proficiency, and length estimations of the curved fascicles of the BF_lh_ using extrapolation methods remain prone to error compared to whole muscle visualization approaches^45^. As the present measures lack test–retest reliability evaluations, we cannot exclude that potential intervention effects on biceps femoris characteristics remained unobserved by random between-session variations.

Nonetheless, interpretations should be considered in relation to the experienced sonographer^31,32,61–64^, and the expected veracity using a manual linear extrapolation method of longitudinal BF_lh_ images from standardized scanning sites. Accordingly, Franchi and colleagues^45^ reported that this approach raises similar BF_lh_ fascicle length estimation compared to extended-field-of-view imaging of the whole fascicle length. Yet, poor within-subject correlations between ultrasound and diffusion tensor MRI of BF_lh_ fascicle length challenge these findings^65^, and it needs further investigations. Second, ultrasound measurements of muscle morphological and architectural characteristics were derived from limited locations of one selected muscle. Adaptive changes after eccentric training may have been better reflected by imaging techniques, such as extended-field-of-view, 3-D ultrasound imaging, or diffusion tensor magnetic resonance imaging, performed over whole muscle groups. Future studies should address such intramuscular or intermuscular adaptations. Third, representative illustrations of Nordic hamstring exercise of previous observations^36,52,66^ and offline analyses of hip flexion during test trials of the present study (average maximum of 14°, see Fig. 4 in Supplementary Dataset) suggest that keeping fully extended hips is challenging, even in trained athletes of high task experience. Potential effects of these hip flexion remain beyond the scope of this study, but this should be taken into account when interpreting our data.

Lastly, in the NHD tests, the forward lean velocity differed (~ 5°∙s^−1^) between the pre and post-test or between groups (Table 2). Although the contraction velocity reportedly affects muscle strength capacities^67^, the present difference in the forward lean velocity is unlikely to have influenced our results. This assumption is supported by our previous^38^ and the present results, with no difference between NHD_max_ and NHD_30_ trials.

## Supplementary Information

Supplementary Information.

## Data Availability

The datasets generated and analysed during the current study are available from the corresponding author on reasonable request.
