# Supplementary material for: Specificity of eccentric hamstring training and the lack of consistency between strength assessments using conventional test devices
Source: Sci Rep. 2021 Jun 28;11:13417. doi: 10.1038/s41598-021-92929-y (PMC8239011; doi:10.1038/s41598-021-92929-y)
Supplement: Supplementary file 1 — Supplementary Information. [file 41598_2021_92929_MOESM1_ESM.pdf]

# SPECIFICITY OF ECCENTRIC HAMSTRING TRAINING AND THE LACK OF CONSISTENCY BETWEEN STRENGTH ASSESSMENTS USING CONVENTIONAL TEST DEVICES

*Hans-Peter Wiesinger, Manuel Scharinger, Alexander Kösters, Christoph Gressenbauer, Erich Müller*
Department of Sport and Exercise Science, University of Salzburg, Salzburg, Austria

**Corresponding Author**
MMMag. Dr. Hans-Peter Wiesinger LLB.oec.
Department of Sport and Exercise Science
Schlossalle 49
5400 Hallein/Rif
Austria
Email: hans-peter.wiesinger@sbg.ac.at
Phone: +4366280444883 / Fax: +43-662-8044-615
ORCID ID
Hans-Peter Wiesinger - 0000-0001-8526-2832

**Eccentric muscle torque and work**

At baseline, eccentric peak torque and average work did not differ between groups (IKD: $F_{(2;27)} \leq .87$, $P \geq .430$, $\eta_p^2 \leq .06$; $NHE_{30}$: $F_{(2;27)} \leq 1.05$, $P \geq .364$, $\eta_p^2 \leq .07$). However, eccentric peak torque was generally higher when measured on the $NHD_{30}$ than the IKD (left: +30%, t=8.12, P<.001, $\eta_p^2$=.69; right: +23%, t=5.76, P<.001, $\eta_p^2$=.53). In contrast, total eccentric work was lower in $NHD_{30}$ than IKD measures (left: -29%, t=5.66, P<.001, $\eta_p^2$=.52; right: -25%, t=6.19, P<.001, $\eta_p^2$=.57).

The time course of the average unilateral torque of each training session is presented in Figure 2a,b. Exercise on the IKD revealed a significant main effect of time ($F_{(1;5)}$=12.84, P<.001, $\eta_p^2$=.59), which was primarily driven by changes after the third week of training. Similarly, the mean torque output on the NHD increased over time (main effect: $F_{(1;5)}$=4.19, P=.003, $\eta_p^2$=.32), with a significant increase in the sixth week (P=.019). There was no effect of leg (main effect: IKD: $F_{(1;5)}$=.04, P<.840, $\eta_p^2$=.05, NHD: $F_{(1;5)}$=.02, P<.901, $\eta_p^2$=.02).

In EPT, significant group × time × device effects (left: $F_{(2;27)}$=9.29, P=.001, $\eta_p^2$=.41; right: $F_{(2;27)}$=10.88, P<.001, $\eta_p^2$=.45) and highly significant main effects for devices (left: $F_{(2;27)}$=90.72, P<.001, $\eta_p^2$=.77; right: $F_{(2;27)}$=55.46, P<.001, $\eta_p^2$=.67) were found. Similar, in eccentric work, significant group × time × device effects (left: $F_{(2;27)}$=12.40, P<.001, $\eta_p^2$=.48; right: $F_{(2;27)}$=7.47, P=.003, $\eta_p^2$=.36) and highly significant main effects for devices (left: $F_{(2;27)}$=40.47, P<.001, $\eta_p^2$=.60; right: $F_{(2;27)}$=50.78, P<.001, $\eta_p^2$=.65) were discovered.

On the IKD, there were significant group × time interactions for the EPT and average work. Changes in EPT were significantly higher in the IG compared to the CG (left: +22%, $Q_{(2,18)}$=4.10, P=.001, $\eta_p^2$=.45; right: +17%, $Q_{(2,18)}$=3.80, P=.002, $\eta_p^2$=.41) and compared to the NG (left: +11%, $Q_{(2,18)}$=3.13, P=.011, $\eta_p^2$=.31; right: +16%, $Q_{(2,18)}$=3.53, P=.004, $\eta_p^2$=.34). There was no significant difference observed in normalised EPT between other groups . Similar, changes in eccentric work were significantly higher in the IG compared to the CG (left: +35%, $Q_{(2,18)}$=6.34, P<.001, $\eta_p^2$=.68; right: +23%, $Q_{(2,18)}$=4.64, P<.001, $\eta_p^2$=.54) and compared to the NG (left: +25%, $Q_{(2,18)}$=4.49, P<.001, $\eta_p^2$=.45; right: +20%, $Q_{(2,18)}$=4.04, P=.001, $\eta_p^2$=.39). No other group differences were found for EPT and average work.

On the NHD, there were significant group × time interactions for the EPT. Post-hoc analysis indicated that changes in EPT were higher in the NG compared to the CG (left: +18%, $Q_{(2,18)}$=3.28, P=.008, $\eta_p^2$=.32; right: +15%, $Q_{(2,18)}$=2.77, P=.026, $\eta_p^2$=.25) and compared to the IG (left: +16%, $Q_{(2,18)}$=2.82, P=.023, $\eta_p^2$=.24; right: +13%, $Q_{(2,18)}$=2.49, P=.048, $\eta_p^2$=.21). There was no significant difference observed in EPT between other groups. Despite significant group × time interactions for the average work, the work produced during the $NHE_{30}$ did not differ between groups. However, there was a trend toward higher values in the NG compared to controls of the left leg (+30%, $Q_{(2,18)}$=2.48, P=.050, $\eta_p^2$=.22).

**NHDmax**

On the NHD, there were some significant group × time interactions for the EPT. Post-hoc analysis indicated that changes in EPT of the left leg were higher in the NG compared to the CG (+17%, $Q_{(2,18)}$=3.35, P=.007, $\eta_p^2$=.31) and compared to the IG (+14%, $Q_{(2,18)}$=2.71, P=.030, $\eta_p^2$=.25). There was no difference observed in EPT for the right leg and between other groups. Changes in eccentric work were significantly higher in the NG compared to the CG of the right leg (+43%, $Q_{(2,18)}$=3.47, P=.005, $\eta_p^2$=.33).

On the NHD, there were some significant group × time interactions for the normalised EPT. Post-hoc analysis indicated that changes in EPT of the left leg were higher in the NG compared to the CG (+18%, $Q_{(2,18)}$=2.71, P=.006, $\eta_p^2$=.33) and compared to the IG (+15%, $Q_{(2,18)}$=3.42, P=.030, $\eta_p^2$=.25). There was no difference observed in EPT for the right leg and between other groups.

**TABLE 1.** Estimation of individual response to isokinetic and Nordic hamstring exercise interventions.

| | Isokinetic Device | | | | Nordic hamstring device | | | |
|---|---|---|---|---|---|---|---|---|
| | IG | | NG | | IG | | NG | |
| | **Left leg** | **Right leg** | **Left leg** | **Right leg** | **Left leg** | **Right leg** | **Left leg** | **Right leg** |
| SWC (%) | 4.01 | 3.84 | 4.01 | 3.84 | 3.07 | 3.13 | 3.07 | 3.13 |
| Mean change (N·m) | 28.2 | 21.5 | 8.4 | 2.5 | 10.0 | 10.5 | 31.7 | 28.3 |
| $SD_{IR}$ (N·m) | 15.1 | 16.0 | 4.2 | 7.2 | 9.3 | -3.3 | 24.4 | 20.6 |
| Proportion of response (%; [CL]) | 94.3 [81-100] | 85.7 [75-98] | 83.1 [44-99] | 39.4 [10-68] | 72.5 [49-97] | 96.9 [54-100] | 86.8 [74-99] | 87.7 [72-100] |
| Standardized mean change (a.u.) | 1.28 | 0.93 | 0.38 | 0.11 | 0.35 | 0.39 | 1.11 | 1.05 |
| Standardized $SD_{IR}$ (a.u.) | 0.69 | 0.70 | 0.19 | 0.31 | 0.33 | -0.12 | 0.85 | 0.76 |

IG, isokinetic group; NG, Nordic group; SCW, smallest worth change; SDIR, standard deviation of individual response; CL, confidence limits of proportion of response (%).

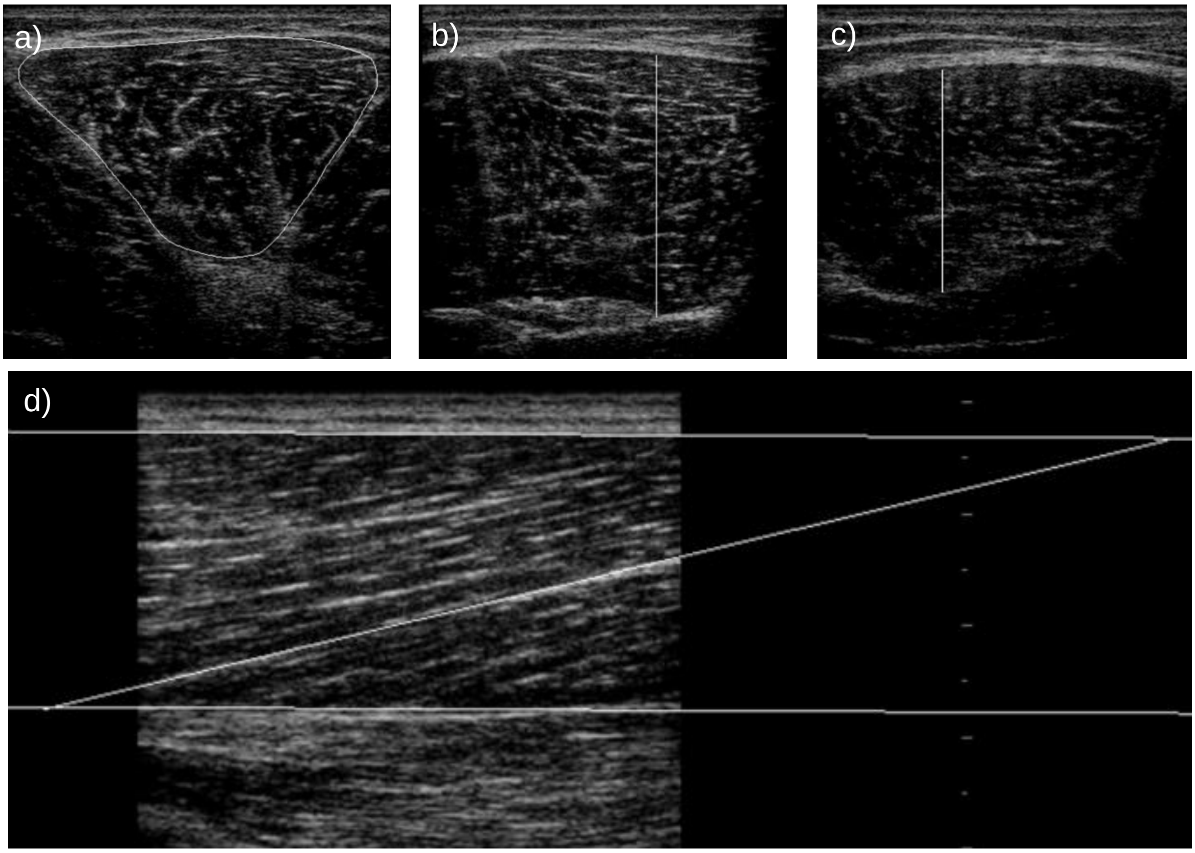


**Figure 1.** Illustration of ultrasound images of biceps femoris long head ($BF_{lh}$) cross-sectional area (a), thickness (b,c) and architecture (d) in one subject. Transversal scan show outlined cross-sectional area at 25% (a), and thickness at 50% (b) and 75% (c) of the $BF_{lh}$. Pennation angle and fascicle length were obtained from sagittal scans at 50% of femur length, at the mid-distance between the lateral and the medial borders of the $BF_{lh}$, as illustrated by one fascicle and superficial and intermediate aponeuroses (d).

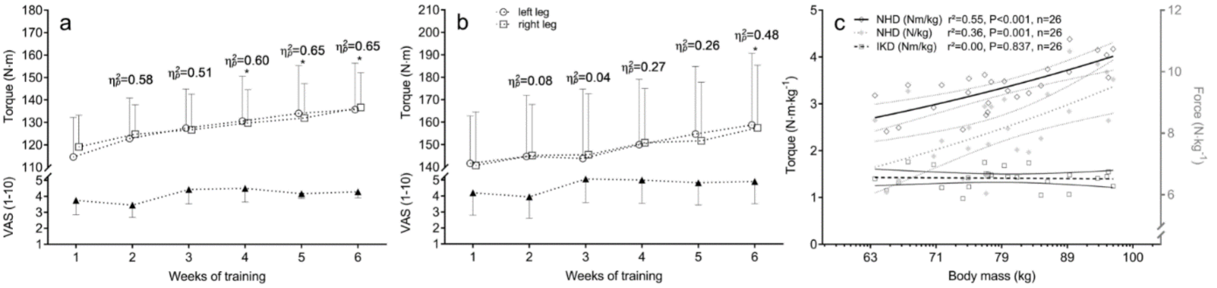


**Figure 2.** Time course of changes in eccentric knee flexor torque and muscle soreness measures on the dynamometer (IKD) (a) and Nordic hamstring device (NHD) (b) and ratio related eccentric knee flexion torque (IKD) and force (NHD) plotted against body mass (c). Average weekly soreness measured using a numeric visual analogue scale (VAS) (1-10) at the beginning of each training session. Values are mean ± 95%CI. * $p<0.05$ (Bonferroni-corrected) versus the average torque of the first intervention week. Data points of body mass were obtained from the baseline characteristics of the recruited subjects. The linear regressions and their 95% confidence limits indicate that body-mass-correlate with the normalised peak eccentric torque (IKD) and normalised peak eccentric force (NHD).

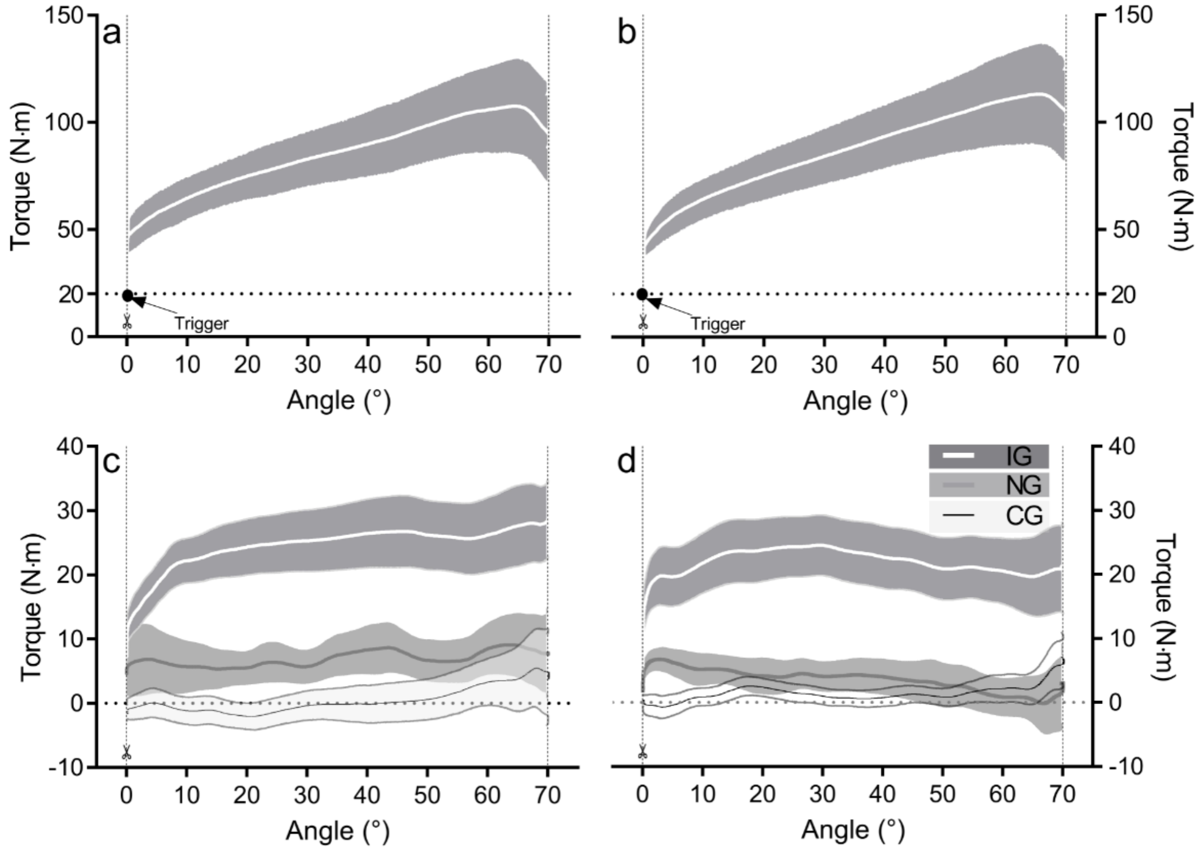


**Figure 3.** Participants pre-intervention isokinetic torque of the left (a) and right leg (a) and changes over the six weeks (c-d).

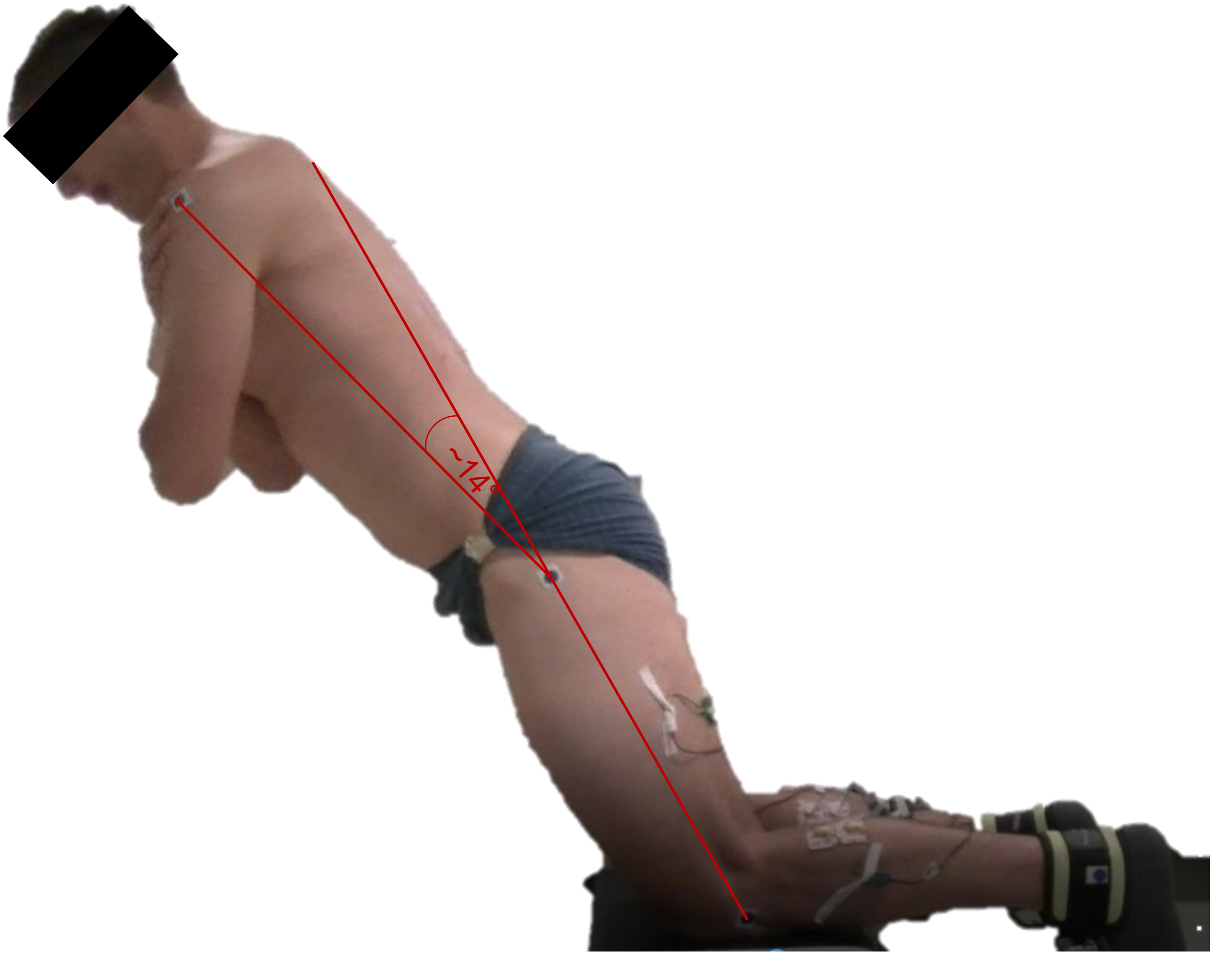


**Figure 4.** Representative illustration of the hip angle.
